# Novel human D-amino acid oxidase inhibitors stabilize an active-site lid-open conformation

**DOI:** 10.1042/BSR20140071

**Published:** 2014-08-11

**Authors:** Ryan T. Terry-Lorenzo, Lawrence E. Chun, Scott P. Brown, Michele L. R. Heffernan, Q. Kevin Fang, Michael A. Orsini, Loredano Pollegioni, Larry W. Hardy, Kerry L. Spear, Thomas H. Large

**Affiliations:** *Discovery Research Department, Sunovion Pharmaceuticals, Marlborough, MA 01752, U.S.A.; †Emerald Bio, Bainbridge Island, WA 98110, U.S.A.; ‡Dipartimento di Biotecnologie e Scienze della Vita, Universita degli Studi dell’Insubria, Via J. H. Dunant 3, 21100 Varese, Italy; §The Protein Factory, Politecnico di Milano, ICRM-CNR Milano and Universita degli Studi dell’Insubria, Via Mancinelli 7, 20131 Milano, Italy

**Keywords:** crystallography, DAAO, DAO, drug discovery, NMDA receptor, schizophrenia, DAAO, D-amino acid oxidase, hDAAO, human D-amino acid oxidase, HRP, horseradish peroxidase, pdb, protein data bank, rDAAO, rat DAAO, NMDAR, *N*-methyl-D-aspartate receptor

## Abstract

The NMDAR (*N*-methyl-D-aspartate receptor) is a central regulator of synaptic plasticity and learning and memory. hDAAO (human D-amino acid oxidase) indirectly reduces NMDAR activity by degrading the NMDAR co-agonist D-serine. Since NMDAR hypofunction is thought to be a foundational defect in schizophrenia, hDAAO inhibitors have potential as treatments for schizophrenia and other nervous system disorders. Here, we sought to identify novel chemicals that inhibit hDAAO activity. We used computational tools to design a focused, purchasable library of compounds. After screening this library for hDAAO inhibition, we identified the structurally novel compound, ‘compound **2**’ [3-(7-hydroxy-2-oxo-4-phenyl-2H-chromen-6-yl)propanoic acid], which displayed low nM hDAAO inhibitory potency (K_i_=7 nM). Although the library was expected to enrich for compounds that were competitive for both D-serine and FAD, compound **2** actually was FAD uncompetitive, much like canonical hDAAO inhibitors such as benzoic acid. Compound **2** and an analog were independently co-crystalized with hDAAO. These compounds stabilized a novel conformation of hDAAO in which the active-site lid was in an open position. These results confirm previous hypotheses regarding active-site lid flexibility of mammalian D-amino acid oxidases and could assist in the design of the next generation of hDAAO inhibitors.

## Abstract 2

Computational tools combined with experimental testing were used to identify novel inhibitors of hDAAO (human D-amino acid oxidase), an enzyme relevant for schizophrenia and neuropathic pain. These inhibitors stabilized a hDAAO conformation with the enzyme's active-site lid open.

## INTRODUCTION

DAAO (D-amino acid oxidase) is a flavoprotein with exquisite stereospecificity for catalysing the oxidation and, hence, degradation of D-amino acids [1,2]. DAAO has been studied for many decades and its catalytic mechanism is well-understood. Using FAD as cofactor, DAAO binds to D-amino acids and, via a hydride transfer mechanism, oxidizes the amino acid and, in the process, reduces the FAD cofactor. DAAO has weak activity towards D-amino acids with acidic side chains, but has significant activity to oxidize a diversity of D-amino acids, typically amino acids with small hydrophilic, large aromatic or basic side chains [1,2].

DAAO is evolutionarily conserved, and in mammals, the significant levels of DAAO protein and enzymatic activity are found in the kidney, liver (some species) and brain [3]. Although the function of DAAO in liver and kidney is likely to degrade D-amino acids originating from bacterial sources [4], the function of DAAO in the mammalian brain was mysterious until the early 1990s, when it was determined that the mammalian brain contains D-amino acids, most notably D-serine [5,6]. Furthermore, brain D-serine exists at concentrations sufficient for functionally serving as a co-agonist at the glycine site of the NMDA (*N*-methyl-D-aspartate) receptor [7–9]. The NMDAR is a glutamate receptor in the brain known to play a central role in the nervous system processes such as synaptic plasticity, learning and memory and pain sensation [10]. This link between D-serine and NMDAR function suggested that, by controlling D-serine brain levels, DAAO and serine racemase, the D-serine-synthesizing enzyme [11], are also involved in regulating the key brain functions. Despite the apparent low levels of DAAO in forebrain regions [12], pharmacological studies with DAAO inhibitors [13,14], genetic studies in DAAO knockout animals [15,16] and genetic studies with serine racemase-mutated mice [17,18] have all clearly indicated that D-serine regulatory enzymes impact NMDAR function, synaptic function and cognitive ability. Furthermore, chemical regulators of D-serine metabolism could be effective disease treatments. More specifically, as NMDAR hypofunction is a core pathway deficit in schizophrenia [19,20], DAAO inhibitors, perhaps in combination with D-serine systemic administration [21], have the potential to be effective treatments of schizophrenia via their capacity to increase D-serine in the brain and enhance NMDAR-dependent functions [13,14,22]. Moreover, although the mechanistic underpinnings of DAAO's roles in nociception are not fully understood, DAAO inhibitors have been shown to reduce pain in various rodent models of neuropathic pain [23–25].

Although multiple academic laboratories and pharmaceutical companies have designed inhibitors of hDAAO (human DAAO) [26], the known collection of potent hDAAO inhibitors is structurally rather limited. Modelled after the classic inhibitor, benzoic acid, the original pharmacophore for hDAAO inhibitors was a low molecular weight, aryl-acid compound that occupied the hDAAO active site on the *re* side of the isoalloxazine ring of FAD [27]. Further efforts revealed that bioisosteric replacement of the acid could produce compounds which were also hDAAO inhibitors with low nM potency [22]. Guided by the knowledge that the active site could accommodate D-amino acids with large aromatic side chains, such as D-tryptophan [28] and D-DOPA [29], a newer generation of hDAAO inhibitors were designed that occupy the so-called ‘subpocket’ of the hDAAO active site [13,30,31]. Additional plasticity in the hDAAO active site beyond the *re* side of flavin and the subpocket has yet to be explored.

hDAAO, unlike other known DAAOs, has low affinity for the FAD cofactor [32], so it likely exists as an equilibrium of FAD-bound (active)- and FAD-unbound (inactive) species in physiological environments [33]. Guided by this knowledge and by the precedented FAD-competitive DAAO inhibitors described in the literature [33–35], we sought compounds that are FAD competitive. To gain specificity for hDAAO inhibition, we sought compounds that combined elements of both the D-amino acid and the flavin portion of the FAD cofactor. Such bisubstrate analogues would be expected to compete with both D-serine and FAD and would represent compounds divergent from existing hDAAO inhibitors. We used computational tools to identify a focused library of bisubstrate analogue-like compounds and screened them for hDAAO inhibition. Serendipitously, however, we discovered a compound that did not compete with FAD, but instead occupied a novel pocket in the hDAAO active site and stabilized an hDAAO conformation with its active-site lid open. The DAAO active-site lid (amino acids 216–228) had previously been hypothesized to open up to allow for substrate access [28]. The X-ray crystal structures described here confirm this hypothesis, extend our knowledge of DAAO active-site flexibility, and enable future opportunities for structure-guided drug design of DAAO inhibitors.

## EXPERIMENTAL

### Compound procurement

The compounds composing the focused library were identified using computational chemistry methods. Briefly, the eMolecules catalogue of commercially available compounds was filtered for acceptable drug-like molecular properties. After filtering, compounds were computationally scored (using both 2D and 3D methods) for their potential to occupy portions of the D-amino acid and FAD-binding pockets within hDAAO. The 1016 best scoring compounds were purchased from eMolecules for screening. Please see Supplementary Online Data (at http://www.bioscirep.org/bsr/034/bsr034e133add.htm) for details on library assembly and screening.

Compound **1** (4H-furo[3,2-b]pyrrole-5-carboxylic acid) was synthesized as described previously [27]. Compound **2** [3-(7-hydroxy-2-oxo-4-phenyl-2H-chromen-6-yl)propanoic acid] was purchased from eMolecules as an original compound from the focused library screen. Compound **3** [4-hydroxy-6-(2-(7-hydroxy-2-oxo-4-phenyl-2H-chromen-6-yl)ethyl)pyridazin-3(2H)-one], Compound **5** (6-(2,4-dihydroxyphenethyl)-4-hydroxypyridazin-3(2*H*)-one) and Compound **6** (6-(2,4-dimethoxyphenethyl)-4-hydroxypyridazin-3(2H)-one) were synthesized and characterized by NMR and MS. Synthesis and analytical details are described in the Supplementary Online Data. ADP disodium salt was purchased from Sigma-Aldrich and Compound **4** was purchased from eMolecules.

### Enzymatic assays

All enzymatic assays were conducted at room temperature (23–24°C) in 96-well plate format. Dose response data to generate IC_50_ data were analysed via Prism (Graphpad Software) or by a script executed by Pipeline Pilot (Accelrys). In each case, data were fit to a standard, four parameter equation to determine curve top, bottom, concentration producing 50% inhibition (IC_50_) and Hill Slope.

An Amplex Red-based assay has been utilized by others to measure hDAAO product formation and screen for hDAAO inhibitors [36]. For our assay, N-terminal hexaHis (His)-tagged hDAAO (prepared as described previously [32]), HRP (horseradish peroxidase), FAD, and compound inhibitor were incubated for 20–30 min. After that pre-incubation period, D-serine and Amplex Red were added and reaction proceeded for 1 hour. Fluorescent product, caused by hydrogen peroxide-dependent, Amplex Red oxidation during hDAAO-catalysed substrate turnover, was measured on a FlexStation II (Molecular Devices) in endpoint mode with these settings: excitation 530 nm, emission 590 nm, cutoff 590 nm and photomultiplier tube on Low. The final concentrations for reaction components were as follows: 50 mM sodium phosphate (Sigma), pH 7.4, 0.001% (v/v) human serum albumin (Sigma), 0.73 nM His-hDAAO (prepared as described in [32]), 4 units/ml HRP (Sigma), 450 nM FAD (Sigma), 50 μM Amplex Red (Life Technologies), 5 mM D-serine (Alfa Aesar) and 1.5% (w/v) DMSO. For the counter assay, hDAAO was omitted and 800 nM hydrogen peroxide (Sigma) was included. For the rDAAO (rat DAAO) assay, the protocol was identical except that hDAAO was replaced with 5 nM hexaHis-tagged rDAAO (prepared as described in [37]) and D-serine concentration was increased to 30 mM.

For the LC–MS-based hDAAO inhibition assay, his-hDAAO, FAD and compound inhibitors were incubated for 20–30 min. After that period, D-phenylglycine was added and reaction proceeded for 1 h. The final concentrations for reaction components were as follows: 50 mM ammonium bicarbonate (Sigma), pH of approximately 8.0 (not adjusted), 0.001% (v/v) human serum albumin, 5 nM His-hDAAO, 450 nM FAD, 5 mM D-phenylglycine (Sigma) and 1% (v/v) DMSO. Following the 1 h enzymatic reaction, an equal volume of 100% (v/v) acetonitrile was added. Quantitation of benzylformic acid concentrations was performed using negative ion liquid chromatography/mass spectrometry/mass spectrometry (LC–MS/MS). A 2 μl aliquot of sample was injected onto an ultra-high performance liquid chromatographic system (UPLC Waters Corp) equipped with an API 5500 QTrap mass spectrometer detector (Applied Biosystems/MDS Sciex) operated in the negative TurboIonSpray® mode. Instrument conditions were adjusted and optimized for benzylformic acid that was monitored using transitions from *m*/*z* 149–77. The separation of benzylformic acid from extracted matrix materials was accomplished with an overall run time of 1.5 min using a Waters Acquity BEH C-18 1.8 μm column (50 mm×2.1 mm) maintained at 25°C. The mobile phases used for elution consisted of 1.0 mM ammonium formate with 0.2% (v/v) formic acid in water (A) and 1.0 mM ammonium formate with 0.2% (v/v) formic acid in acetonitrile (B) at a total flow rate of 0.600 ml/min. Wash solvent 1 was 3% formic acid in acetonitrile and wash solvent 2 was 3% formic acid in water. Calibration standards were injected once before and once after the analysis of unknown samples to construct a standard curve. A linear weighted (1/concentration^2^) regression analysis of the analyte peak area ratio versus theoretical concentration was used to produce calibration curves from standards.

A jump-dilution protocol [38] was utilized to confirm reversibility of compound inhibition and to determine compound apparent dissociation rate (k_off_). The assay mixture was similar to that described above for the Amplex Red-based assay system. For the jump-dilution assay, in 5 μl, 15–40 nM hDAAO was incubated with inhibitor compound at a high concentration (typically 6-fold higher than the IC_50_) in the presence of 80 μM FAD. As all the compounds tested were FAD uncompetitive, the high [FAD] facilitated inhibitor–hDAAO complex formation. After a 30 min pre-incubation to form inhibited complexes, 195 μl of reaction mixture was added. Compared with the standard assay, 50 mM D-serine was utilized as the hDAAO substrate. With the 40-fold dilution into high-substrate concentration, after dissociation, compound re-association with hDAAO would be unlikely and marginal, as the diluted compound concentration would be well below an effective inhibitory concentration. Immediately after adding the reaction mixture, fluorescent substrate was monitored kinetically by the FlexStation II. Data were fit using the following equation [38] in which *P*is the fluorescent product formed, *t*is the time, *v*_s_ is the final, steady-state reaction velocity, *v*_i_ is the initial reaction velocity, and *k* is the *k*_off_:

(1) $$\left[P\right]=v_{s}t+\frac{v_{i}-v_{s}}{k}\left(1-e^{-\mathrm{kt}}\right)$$

To determine inhibitor mechanism of action (D-serine- or FAD-competition), saturation experiments were performed using the Amplex Red system. Keeping D-serine or FAD constant, concentration of the other substrate was varied, and hDAAO enzymatic product was measured fluorescently as described above; these saturation tests were conducted in the presence of variable amounts of inhibitor. In these experiments, there was no preincubation period, such that hDAAO was exposed to D-serine, FAD and inhibitor simultaneously. The final concentrations for reaction components were as follows: 50 mM sodium phosphate, pH 7.4, 0.001% (v/v) human serum albumin, 0.15 nM hDAAO, 4 units/ml HRP, 40 μM FAD (constant for D-serine saturation), 50 μM Amplex Red, 50 mM D-serine (constant for FAD saturation), and 1.5% DMSO. After a 1 h reaction, product was measured using the FlexStation II in the endpoint mode. Data were plotted and fit with a one site, specific binding equation (Graphpad Prism):

(2) $${y=V}_{\max}\times \frac{x}{K_{M}+x}$$

Equation (2) permitted a determination of *V*_max_ and *K*_M_ at variable inhibitor concentration for a determination of competitive, uncompetitive or non-competitive profile. For D-serine competitive inhibitors, the same data were fit using the GraphPad Prism ‘Competitive inhibition’ equation to derive a global K_i_ value:

(3) $$K_{M,\mathrm{obs}}=K_{M}\left(1+\left[I\right]K_{i}\right)$$

(4) $$y=V_{\max}\times \frac{x}{K_{M,\mathrm{obs}}+x}$$

### Crystallization and structure determination

Full-length, untagged hDAAO protein was prepared as described previously [32]. The hDAAO preparation at 2 mg/ml was dialysed overnight at 4°C against 50 mM sodium phosphate, pH 6.6 and 10 μM FAD prior to crystallography. hDAAO samples were incubated overnight with 250 μM compound **3** or 1 mM compound **2** and then subjected to crystallization trials. Crystals of the compound **3** complex were grown in sitting drops containing 0.4 μl hDAAO+0.4 μl crystallant consisting of 30% (w/v) PEG2000MME, 0.1 M potassium thiocynate. Crystals of the compound **2** complex were grown in sitting drops containing 0.4 μl hDAAO+0.4 μl crystallant consisting of 13.64% (w/v) PEG3350, 0.1 M Tris pH 7.4, 0.15 M potassium citrate tribasic. Crystals were harvested in 20% (v/v) ethylene glycol and flash frozen in liquid nitrogen. X-ray data for the hDAAO:FAD:compound **2** complex crystals were collected at SSRL beam line 7-1 and reduced with the XDS/XSCALE package to 2.85 Å. The CCP4 program Phaser was used for Molecular Replacement using chain A from the 3CUK structure as the search model. The model was iteratively extended in real space using Coot and refined in reciprocal space using Refmac5. The final model had R/R_free_=0.24/0.29. X-ray data for the hDAAO:FAD:compound **3** complex crystals were collected at APS beam line 21-idf and reduced with the XDS/XSCALE package to 2.4 Å. The CCP4 program Molrep was used for Molecular Replacement using chain A from the 3CUK structure as the search model. The model was iteratively extended in real space using Coot and refined in reciprocal space using Refmac5. The final model had *R*/*R*_free_=0.18/0.23.

## RESULTS

### Characterization of compound 2 as a novel inhibitor of hDAAO

**Figure 1:**
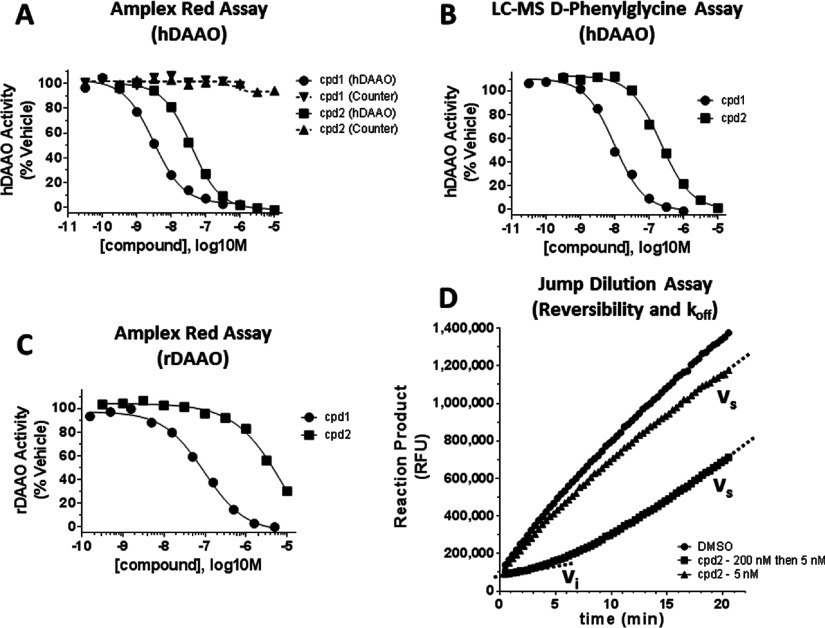
Profiling compounds in enzymatic inhibitory assays (**A**) In the Amplex Red platform, compound (cpd) **1** and cpd **2** were tested for hDAAO inhibition with D-serine as substrate (solid lines) and counter assay inhibition (dashed lines). Neither compound produced any inhibition in the counter assay. (**B**) Cpd **1** and cpd **2** inhibited hDAAO in the hDAAO inhibition assay with D-phenylglycine as substrate and using LC–MS for direct product detection. (**C**) Cpd **1** and cpd **2** inhibited rDAAO, albeit less potently than they inhibited hDAAO. (**D**) Jump-dilution assay example data for cpd **2**. In the Amplex Red platform, activity of hDAAO was monitored kinetically as production of fluorescent product over time. In the ‘k_off_’ condition (squares; ‘200 nM then 5 nM’), 200 nM cpd **2** (an inhibitory concentration; see Figure 2A) was rapidly diluted to 5 nM (a non-inhibitory concentration). Initially hDAAO was inhibited as reported by a shallow slope of product production defined as low initial velocity (*v*_i_). Over time, inhibitor dissociates and a more rapid, steady-state velocity of product production (*v*_s_) was observed. Quantitative data from each of these assays are presented in Table 1.

We utilized a novel screening strategy to identify compound **2**, a potent inhibitor of hDAAO (See Supplementary Online Data; compound **2** structure in Table 1). To confirm that compound **2** was a reversible inhibitor of hDAAO, we profiled it in a series of biochemical assays, using the previously characterized hDAAO inhibitor compound **1** [22,27] as positive control. As shown in Figure 1(A), compounds **1** and **2** inhibited hDAAO activity. However, they did not display inhibition in the counter assay, verifying that these compounds did not interfere with the assay detection system non-specifically. To fully exclude fluorescence artefacts that could arise in the Amplex Red system, we developed an orthogonal assay in which the direct product of the hDAAO oxidative reaction was measured by LC–MS, when D-phenylglycine was used as substrate. In this assay, both compounds **1** and **2** were confirmed as potent hDAAO inhibitors (Figure 1B and Table 1). Both compounds inhibited rDAAO, although the compounds were less potent as inhibitors of rDAAO compared with hDAAO (Figure 1C and Table 1). Compound **1** and other hDAAO inhibitors have previously been observed to be less potent rDAAO inhibitors [22]. To confirm that the compounds were reversible, we utilized a jump-dilution assay protocol, in which the inhibited enzyme–ligand complexes were diluted to allow for kinetic measurement of inhibitor dissociation and reconstitution of enzyme activity [38]. For compound 2, we pre-incubated in 200 nM (a concentration yielding >80% inhibition; see Figure 1A), and then after dilution the compound was at 5 nM (no significant inhibition under 50 mM D-serine recovery conditions). Reaction product was recorded immediately after dilution (Figure 1D). Under these conditions, initial velocity (*V*_i_) was low, but over time, inhibitor dissociated and a steady-state velocity (*V*_s_) was reached. These results were consistent with reversible inhibition [38]. The recovery time course was fit with equation (1) (the Experimental section) to obtain an apparent rate constant for dissociation (*k*_off_) as reported in Table 1.

**Table 1:** Summary of the inhibitory properties of hDAAO inhibitors All data are presented as mean value±S.D. (number of separate experiments in parentheses). N.D.=not determined. The four assays for which IC_50_ data were generated were conducted with either hDAAO or rDAAO. Assays were conducted either in the Amplex Red format (Amplex) or by direct product detection using LC–MS. The K_i_ values were determined by fitting data from the saturation experiments to equations (3) and (4) (see Figure 2 and the Experimental section).

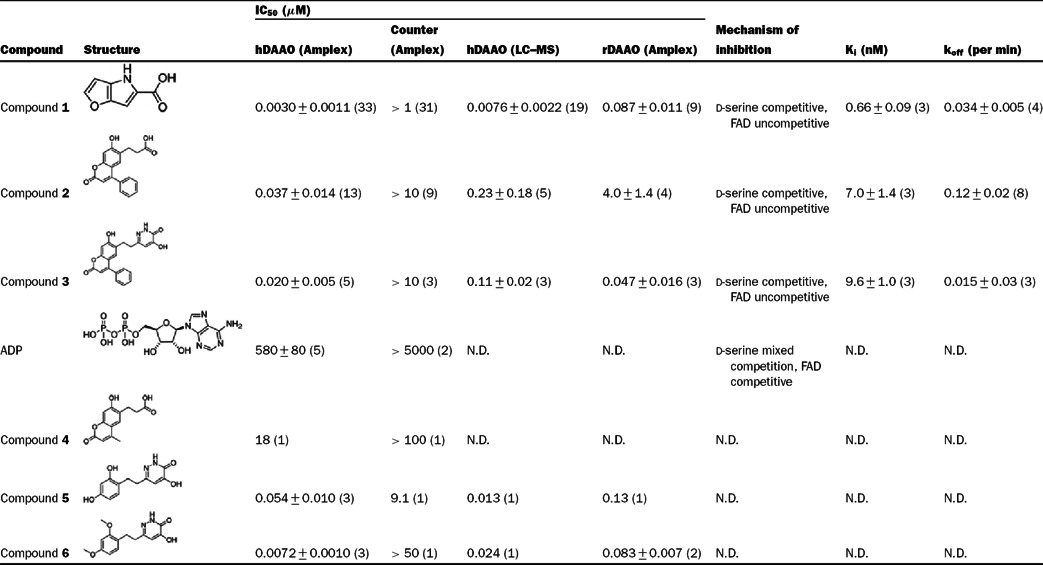

### Compound 2 had the kinetic profile of an active-site hDAAO inhibitor

**Figure 2:**
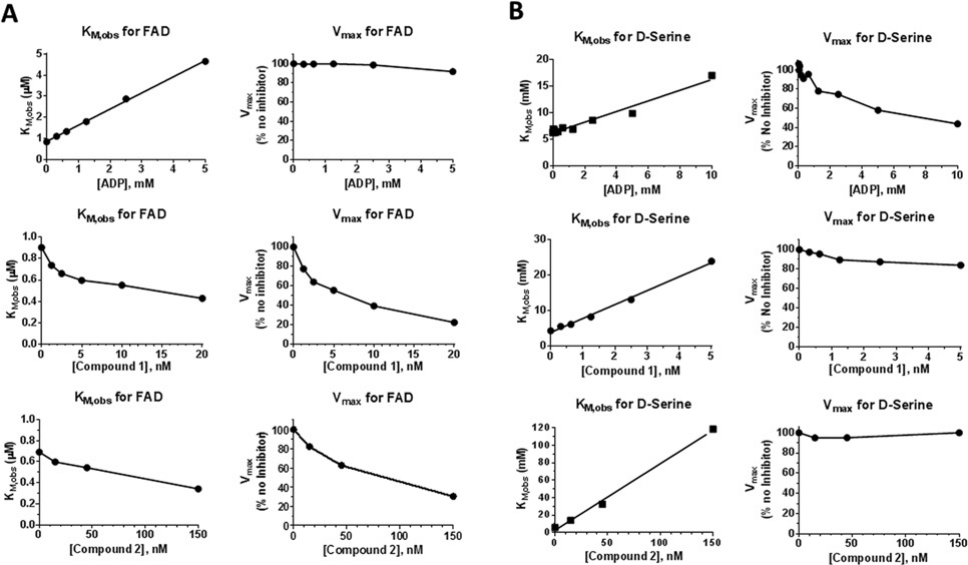
Determination of compound kinetic behavior in FAD- and D-serine saturation experiments (**A**) Under conditions of constant, high (50 mM) D-serine concentration, FAD saturation experiments were performed. Using non-linear curve-fitting, *K*_M_,_obs,FAD_ (left) and *V*_max_ (right) for FAD were determined at variable concentrations of tested inhibitor. (**B**) Reciprocally to Figure 2(A), under conditions of constant, high (40 μM) FAD concentration, D-serine saturation experiments were performed to obtain *K*_M_,_obs,D-ser_ (left) and *V*_max_ (right) values at variable concentrations of tested inhibitor.

As described in the Supplementary Online Data section, compound **2** was hypothesized based upon ligand-based and structure-based computer modelling to occupy both the active site of hDAAO and portions of the FAD-binding site. To experimentally determine competitive behaviour of compound **2** with both substrate (D-serine) and cofactor (FAD), we tested it in D-serine and FAD saturation experiments. To confirm that our assay was capable of reporting FAD-competitive kinetic behaviour, we profiled a series of molecules that we hypothesized would be FAD competitive. One of these compounds was ADP, a molecule identical to a substructure of FAD; the ADP portion of FAD sits deep into the hDAAO protein [39]. ADP was a low-potency hDAAO inhibitor (Table 1) that we hypothesized would displace FAD and behave kinetically as FAD-competitive. In FAD saturation experiments, ADP linearly increased hDAAO K_M_,_obs_ for FAD with minimal effect on hDAAO V_max_ (Figure 2A, top). This result is consistent with ADP being an FAD-competitive inhibitor of hDAAO and confirms that our assay could report FAD-competitive behaviour. In reciprocal D-serine saturation experiments, ADP displayed a mixed competition profile in which it both lowered V_max_ and increased K_M_,_obs_ (Figure 2B, top). Of note, when the active site of hDAAO is occupied by a ligand, hDAAO affinity for FAD increases [32,33]; thus, even a ‘pure’ FAD competitive inhibitor would be expected to appear partially D-serine competitive in these experiments.

In contrast to ADP, compound **1**, which occupies the hDAAO active site and is stabilized by pi (π–π)-stacking interactions with the FAD cofactor [27], dose-dependently decreased both the V_max_ and K_M_,_obs_ for FAD (Figure 2A). This uncompetitive kinetic behaviour is consistent with compound **1** binding only to the FAD-bound, hDAAO holoenzyme [27]. In saturation experiments with D-serine, compound **1** displayed classic competitive behaviour: the value of *K*_M_,_obs_ for D-serine increased linearly with increasing concentration of compound **1**, whereas *V*_max_ was only modestly affected (Figure 2B). In summary, in our experiments, the hDAAO active-site inhibitor compound **1** was D-serine competitive and FAD uncompetitive. For D-serine competitive compounds, kinetic equations (3) and (4) (see the Experimental section) were used to derive an inhibitory constant (*K*_i_), as reported in Table 1. For each compound, the *K*_i_ was lower (more potent) than the IC_50_ value, which was likely owing to the variable concentrations of FAD and D-serine used in the two assay formats.

Compound **2** was tested in these assays and determined to be both FAD uncompetitive (decreased FAD *K*_M_,_obs_ and *V*_max_, Figure 2A) and D-serine competitive (increased hDAAO *K*_M_,_obs_ for D-serine without affecting *V*_max_, Figure 2B). This result demonstrated that, inconsistent with the modelling predictions, compound **2** did not compete with FAD. Rather, despite its larger molecular mass relative to previously reported active-site hDAAO inhibitors [13,22,27,30,40], compound **2** displayed the kinetic properties of a canonical, active-site-bound hDAAO inhibitor.

### Compound 2 bound to hDAAO in an active-site lid-open conformation

**Figure 3:**
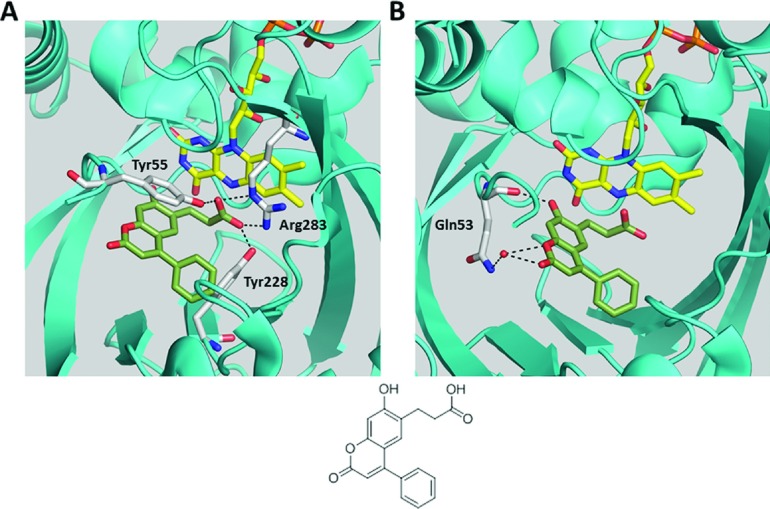
Cpd 2 interacts with the active site of hDAAO In this figure and in subsequent crystallography images, illustrated amino acid side chain carbons are uncoloured, carbons of FAD are yellow, and ligand carbons are differentially coloured. Compound structures of ligands included in the crystallographic images are displayed at the bottom of the images. (**A**) In the X-ray crystal structure, cpd **2** (green) binds in the active site of hDAAO adjacent to the FAD cofactor. The carboxylic acid moiety of cpd **2** forms four different hydrogen bonds (dashed lines) with Arg^283^, Tyr^55^ and Tyr^228^ of hDAAO. The aromatic ring of the Tyr^55^ side chain also forms a π–π stacking interaction with the coumarin of cpd **2** (π–π stacking interactions not shown for image clarity). (**B**) Unlike other hDAAO inhibitors described in the literature, cpd **2** forms hydrogen bonds with the carbonyl backbone of hDAAO Gln^53^ and forms a hydrogen bond with a water molecule (red sphere).

**Figure 4:**
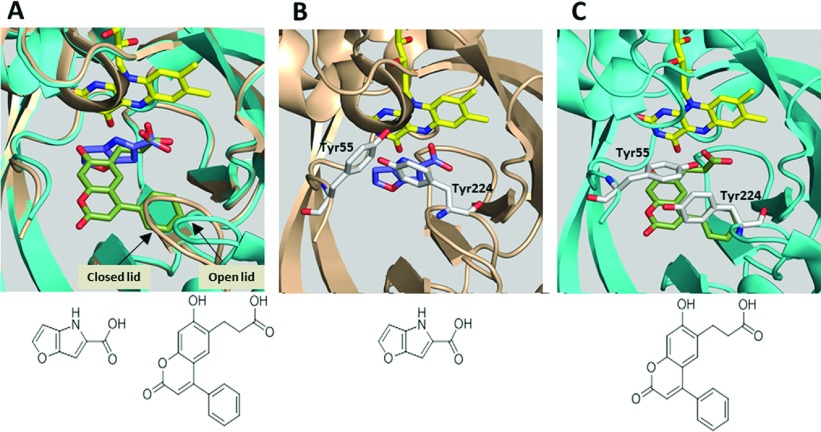
Cpd 2 binds to hDAAO in an active-site lid-open conformation Overlay of the previously published (pdb ID: 3CUK [27]) cpd **1**–hDAAO structure with the cpd **2**–hDAAO structure. In all panels, the cpd **1**–hDAAO protein backbone is brown and the cpd **2**–hDAAO protein backbone is teal. Cpd **1** carbons are blue and cpd **2** carbons are green in all images. (**A**) In the overlay, the primary distinctive movement of the hDAAO backbone is the active-site lid (a loop comprised amino acids 218–224). Relative to the hDAAO–compound **1** structure, this loop moves away from the hDAAO active site when hDAAO is bound to cpd **2**. The cpd **2**-induced hDAAO conformation is defined as ‘active-site lid open,’ consistent with past publications [28]. In these panels, solvent exposure and, hence, substrate access is down and towards the viewer, just past the active-site lid. (**B**) In the active-site lid closed conformation of hDAAO with cpd **1**, Tyr^224^, a part of the active-site lid, is in proximity to the ligand. (**C**) In the active-site lid-open conformation of hDAAO with cpd **2**, Tyr^224^ moves away from the ligand, and the Tyr^55^ side chain rotates towards the ligand.

The binding mode of compound **2** to the hDAAO:FAD holoenzyme was determined by X-ray crystallography (crystallographic data in Table 2). In agreement with our kinetic observations (Figure 2), compound 2 occupied the active site of hDAAO adjacent to the FAD cofactor. The binding interaction was consistent with previously published cocrystal structures of hDAAO with small aryl carboxylic acid inhibitors or carboxylic acid bioisosteres [13,22,27,30] and included a bidentate hydrogen bond from the compound propanoate arm to the guanidinium group of Arg^283^ (Figure 3A). This propanoic acid-binding interaction was further stabilized by hydrogen bonds with Tyr^228^ and Tyr^55^ (Figure 3A). The Tyr^55^ interaction was only available following local rearrangement of the side chains of the active site because of inhibitor binding. This interaction with Tyr^55^ was not seen in past hDAAO–ligand cocrystal structures (for example, see Figure 4B). Additional interactions not observed in previous hDAAO–ligand structures included a hydrogen bond between the coumarin hydroxyl and the carbonyl backbone of Gln^53^ and a water molecule interaction with the cyclic oxygen and carbonyl of compound **2**'s coumarin ring (Figure 3B).

**Table 2:** Crystallographic data

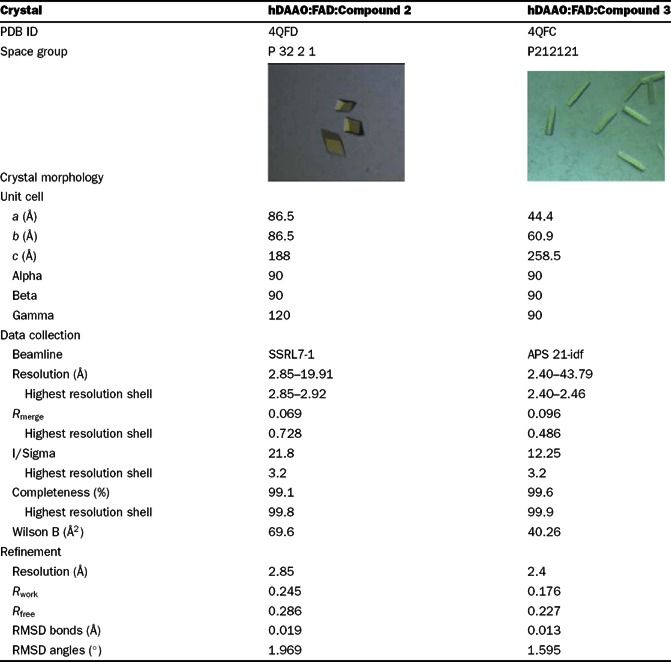

The flexible propanoate arm allowed the coumarin portion of compound **2** to extend at 90° relative to the face of the flavin ring into the centre of the ligand-binding site. This active-site area, also termed a ‘subpocket’ [30], is known to associate with the bulky side chains of DAAO substrates such as D-tryptophan [28] and is occupied by portions of some DAAO inhibitors [13,30]. The hDAAO-compound **2** structure demonstrated that this subpocket is even more flexible than previously envisioned; it accommodates conformational rearrangements that allow the phenyl group on the 4 position of the coumarin ring to occupy a previously undefined pocket in hDAAO. This new hDAAO pocket was generated by the movement of amino acids 218–224, part of the active-site lid of hDAAO [28], away from the inhibitor pocket, allowing the compound **2** phenyl group to occupy space previously occupied by Tyr^224^ (Figure 4). The movement of Tyr^224^ away from the active-site pocket allowed Tyr^55^ to assume a rotamer conformation extending into the pocket where it was stabilized by π–π-stacking interactions with the coumarin heterocycle and hydrogen-bonding interactions with Arg^283^.

Similar to substrates [28,29], but unlike other high-affinity hDAAO ligands [13,22,27,30], no portion of compound **2** π–π-stacked with the isoalloxazine ring of FAD. Also unlike other high-affinity hDAAO ligands, there was no suitable hydrogen-bond donor in compound **2** to interact with the carbonyl backbone of Gly^313^ in hDAAO.

### Compound 3 also bound to hDAAO in an active-site lid-open conformation

As hDAAO is a brain target relevant for schizophrenia [26], and because carboxylic acids generally restrict compounds from entering the brain [41], we sought to replace the compound **2** carboxylic acid with a bioisostere which could maintain the interaction with Arg^283^. After synthesizing and testing several analogues of compound **2**, we discovered compound **3**, in which the carboxylic acid moiety of compound **2** was replaced with a hydroxyl–pyridazinone group (structures in Table 1). Profiling of compound **3** in our panel of assays demonstrated that it was more potent than compound **2** in each of the assays, with a particular improvement in rDAAO inhibition (Table 1). In addition, it had a slow off-rate (0.015 min^−1^), which was even slower than the dissociation of the more potent compound 1 (0.034 min^−1^) (Table 1). Therefore compound **3** is a relatively slow on–off compound that forms a stable, but reversible interaction with hDAAO.

**Figure 5:**
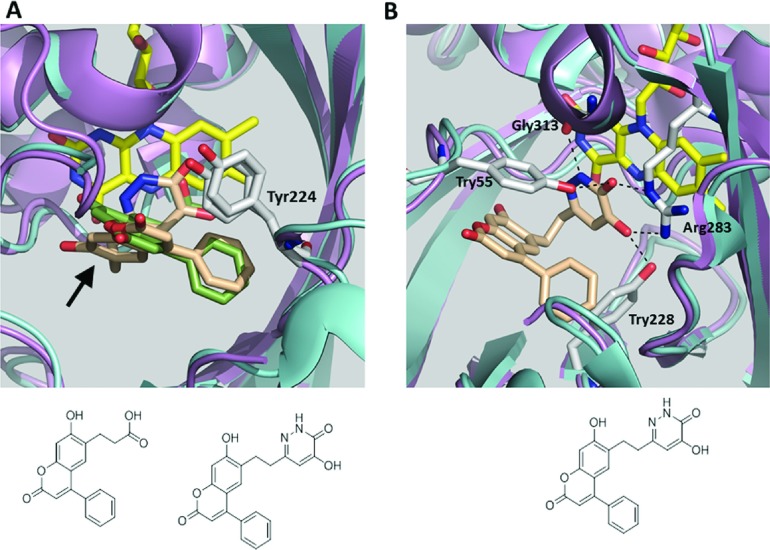
Cpd 3 binds to hDAAO in an active-site lid-open conformation In these panels, the carbons of cpd **2** are green and compound **3** carbons are tan. (**A**) An overlay of the active-site structures of hDAAO in complex with cpd **2** and cpd **3**, oriented such that the viewer is looking down into the hDAAO subpocket. The hydroxyl–pyridazinone group of cpd **3** overlaps with the carboxylic acid of cpd **2**. The phenyl groups of both compounds also overlap. Relative to cpd **2**, cpd **3** has a tilted path through the hDAAO active-site subpocket (arrow). In each structure, Tyr^224^ and associated peptide backbone is tilted away from the active-site, consistent with the active-site lid-open conformation. (**B**) The hydroxyl–pyridazinone group of cpd **3** is an effective carboxylic acid bioisostere, forming hydrogen bonds with Tyr^55^, Arg^283^ and Tyr^228^. These are the same interactions formed by the carboxylic acid moiety of cpd **2** (Figure 3A). The N on the pyridazinone ring forms a hydrogen bond with the carbonyl in the backbone of hDAAO Gly^313^. Although not shown for clarity, the carbonyl moiety on the cpd **3** coumarin ring forms additional hydrogen bonds with a water molecule and with an N–H from the hDAAO backbone at Leu^56^.

The binding mode of compound **3** to the hDAAO:FAD holoenzyme was determined by X-ray crystallography. In this case, a higher resolution crystal form (P2_1_2_1_2_1_) was identified through sparse matrix screening and the structure resolved to 2.4 Å (Table 2). Superimposition of the compound **2** and compound **3** structures [RMSD (root-mean-square deviation)=0.543 Å (301–301 atoms)] showed an overall similar binding pose within the hDAAO active-site pocket (Figure 5A). The ketone and hydroxyl groups of compound **3** formed hydrogen bonds with Arg^283^ (Figure 5B), demonstrating that the hydroxyl–pyridazinone was an effective bioisosteric replacement of the carboxylic acid. The hydroxyl–pyridazinone substituent was stabilized in the hDAAO pocket by a total of five hydrogen bonds, two with Arg^283^, one with the Tyr^228^ hydroxyl, one with the Tyr^55^ hydroxyl, and one with the backbone carbonyl of Gly^313^ (Figure 5B). On the other end of the molecules, the phenyl groups of compounds **2** and **3** both occupied the same activesite lid-open space in hDAAO. In the compound **3**–hDAAO structure, amino acids 219–222 were not included in the refined structure because of a lack of defined electron density. However, we defined the compound **3**–hDAAO structure as active-site lid open based upon two points of evidence: (1) The phenyl group of compound **3** overlaid with the phenyl group of compound **2** and would have sterically clashed with a closed active-site lid. (2) Movement of the Tyr^224^ backbone and side chain of hDAAO was nearly identical in both compounds **2** and **3**–hDAAO structures (Figure 5A), confirming that at least this portion of the lid moves away from the ligand. As compound **3** is a slightly larger molecule than compound **2**, to accommodate this ligand, compound **3** had a ‘tilt’ through the subpocket of the active site (arrow in Figure 5A). This path through the active-site subpocket was observed in a prior crystal structure of hDAAO bound to a different ligand [SEP-137 in [13]; protein data bank (pdb) ID: 3ZNO].

### Analogues of compounds 2 and 3 explore the importance of the active-site lid open conformation

To explore the importance of the active-site lid open conformation, we tested compound **4** for hDAAO inhibitory potency. As shown in Table 1, compound **4**, which is a compound **2** analogue lacking the phenyl group that occupied the active-site lid-open pocket, was a poor hDAAO inhibitor with potency approximately 500-fold weaker than compound **2**. This result suggests that the active-site lid-open conformation facilitated binding of compound **2**. We also tested two synthetic intermediates of compound **3** (see Supplementary Figure S1; available at http://www.bioscirep.org/bsr/034/bsr034e133add.htm). As shown in Table 1, these two compounds, compounds **5** and **6,** displayed potency in several of our biochemical assays that was similar to the potency of compound **3** (IC_50_ values within 5–10-fold). This result, unsurprising based upon data in the literature-testing compounds very similar to compounds **5** and **6** [30], suggests that for these hydroxyl–pyridazinone-containing compounds, the movement of the active-site lid to an open conformation did not enhance inhibitor potency.

## DISCUSSION

In an effort to identify novel hDAAO inhibitors, we used computational tools to identify commercially available, putative bisubstrate analogue-like compounds. We screened a library of approximately 1000 of these compounds for hDAAO inhibition. The most potent hit from that screen, compound **2**, had a structure unlike existing hDAAO inhibitors. Although our library was designed to select compounds that would be both D-serine and FAD-competitive, kinetic experiments demonstrated that compound **2** was actually FAD uncompetitive. Consistent with this observation, the ternary co-crystal structure of hDAAO with compound **2** and FAD demonstrated that compound **2** fit into the hDAAO active site adjacent to FAD. The bulky, aromatic portion of compound **2** occupied the hDAAO subpocket and stabilized an opening of the active-site lid. This finding opens the possibility of discovering structurally more diverse DAAO ligands.

Compound **2** interacts with hDAAO via several interactions that are not seen in other hDAAO inhibitors. The most striking feature is the movement of amino acids 218–224 (a portion of the active-site lid) several angstroms away from the rest of the hDAAO active site (Figure 4A). This backbone movement clears Tyr^224^ away from the active site. As Tyr^224^ is thought to act as a gatekeeper, controlling compound/substrate access to the subpocket [13,28,30], the major backbone movement facilitates access of compound **2** to the subpocket region. An amino acid side chain movement facilitated by the Tyr^224^ retreat is the rotation of Tyr^55^ towards compound **2**, forming π–π stacking and hydrogen bonds with compound **2**. Other novel features of the compound **2**-hDAAO structure are hydrogen bonds between the coumarin bicyclic ring of compound **2** with water and hDAAO (Figure 3B). This new structure is also interesting because of what it lacks. High-affinity hDAAO inhibitors reported by Merck [27], Pfizer [22], Johns Hopkins Brain Science Institute [40], Astellas [30], and ourselves [13] typically have two features in common: (1) a hydrogen-bond donor-oriented towards the carbonyl of hDAAO Gly^313^, and (2) an aromatic ring that can π–π stack with the isoalloxazine ring in FAD. Although some high-affinity hDAAO inhibitors lack interaction with hDAAO Gly^313^ (for example, compound 5 from [13]; pdb ID: 3ZNP), these compounds still are stabilized by the π–π stack with FAD. Because compound **2** is a high-potency hDAAO inhibitor (*K*_i_=7 nM) which lacks both of these two features, the novel interactions of compound **2** with hDAAO are presumably strong enough to make up for loss of these two interactions.

Guided by high-affinity ligands that contain a hydroxyl-pyridazinone group [30], we designed compound **3**. In our compound scaffold, hydroxyl-pyridazinone was an effective bioisosteric replacement for the carboxylic acid of compound **2**. Indeed, compound **3**, unlike compound **2**, displayed the two modes of interaction with hDAAO discussed above: a hydrogen bond with Gly^313^ of hDAAO and a π–π stacking interaction with FAD (Figure 5B). Compound **3** was approximately 2-fold more potent as a hDAAO inhibitor, with enhanced potency driven largely by a particularly slow *k*_off_ (Table 1). However, this rather modest potency boost after gaining two additional major hDAAO interactions suggest that the path through the subpocket by compound **3** is less favourable than for compound **2** (Figure 5A), or that some of the interactions found in compound **2** but not in compound **3** (see Figure 3B for two examples) are key drivers of binding affinity and, hence, inhibitory potency.

This study enables us to comment on the rigidity or flexibility of various regions of the hDAAO active site. In all hDAAO substrate and inhibitor complexes examined, including the compounds reported here, Arg^283^ and FAD appear inflexible. This rigidity could be expected as the Arg^283^–carboxylic acid association in substrates is critical to position the substrate in the correct orientation on the *re* face of the flavin portion of the FAD cofactor to facilitate oxidation [2]. More distant from the precise site of the oxidative reaction, the hDAAO active site appears to be more flexible. The region termed the subpocket [30] (occupied by the coumarin ring in compounds **2** and **3**), has demonstrated flexibility in past structures, particularly in rotamer movements of Tyr^224^ [13,28,30]. In this study, with the hDAAO backbone movement causing a several angstrom Tyr^224^ movement away from the active site, additional flexibility in the subpocket is revealed. This can be observed most clearly by the different routes ligands traverse through this region (e.g. Figure 5A). Finally, the active-site lid (a loop formed by amino acids 216–224) may be a region of extensive flexibility. We did not observe electron density for the full active-site lid in the compound **3**–hDAAO structure, indicating that this flexibility is associated with structural heterogeneity. Additional structural information will be required to make firm conclusions about the various conformations that can be adopted by this region of hDAAO. The flexibility/adaptability of the DAAO active site correlates well with its broad substrate specificity: the rearrangement of residue side chains located on the *re* side of the flavin isoalloxazine ring allows DAAO to bind D-amino acids with significantly different size, including unnatural ones such as cephalosporin C and naphthyl-amino acids [42,43].

The active-site lid of DAAO has long been suspected of being a mobile region of the protein, which must open to allow entry of D-amino acids into the DAAO active site [28,44]. Following substrate entry, the closed lid conformation of DAAO gene-rates a hydrophobic environment that contributes to DAAO substrate specificity [45] and facilitates hydride transfer during substrate oxidation [28]. To date, all structures of yeast, pig, and human DAAO, whether in the substrate-bound, inhibitor-bound, or unbound forms, have portrayed DAAO with this lid closed. Thus, the structures reported here are revealing in that, by capturing hDAAO in a lid-open conformation, they provide direct evidence which confirms past hypotheses of lid flexibility [28,44]. Compounds **2** and **3** are potent hDAAO inhibitors with K_i_ values<10 nM and slow off-rates (for example, compound **3** has a dissociative half-life of nearly 1 h; Table 1). Thus, this ligand-induced, active-site lid-open conformation is an alternative, stable conformation of hDAAO. Although the lid-open conformation is stable, it is revealing that the non-substrate-bound form of hDAAO has only been crystalized in the closed conformation [29]. This result suggests that, although the active-site lid can sample at least two distinct conformations, the closed form is presumably a more favourable, lower free-energy state. As seen with compounds **4**, **5** and **6** (Table 1), the lid-open conformation has variable influence on inhibitor potency. In the compound **2** scaffold, loss of the phenyl group that occupies the active-site lid-open pocket greatly reduced potency and, presumably, affinity (compound **4** in Table 1). However, in the hydroxyl–pyridazinone compound series, the additional interactions afforded by the lid-open conformation did not enhance inhibitory potency (compare compound **3** with compounds **5** and **6** in Table 1). Further experiments are needed to explore the energetic favourability of the active-site lid-open versus -closed conformations of hDAAO. As a final consideration of the relevance of the active-site lid-open conformation, because compound **3** is a potent rDAAO inhibitor (Table 1), this lid-open conformation is likely to exist in DAAO proteins from non-human species as well.

Multiple groups have pursued the discovery of potent, brain-penetrant, drug-like hDAAO inhibitors for use in treating schizophrenia and other nervous system disorders [26]. Thus, the identification of a novel hDAAO conformation could guide the design of the next generation of hDAAO inhibitors. The structures described herein reveal a lid-open space for the design of DAAO inhibitors and show that π–π stacking interactions with FAD are not required for potent inhibition. As we expected, in *in vivo* studies in which compound **2** or **3** were dosed intraperitoneally in mice, each compound displayed low brain penetration (brain:plasma compound ratios <0.05; results not shown). Like all existing high-affinity hDAAO inhibitors, interaction of these compounds with Arg^283^ of hDAAO requires a carboxylic acid or other electronegative moiety, a feature that is known to reduce brain penetration [41]. Therefore we hypothesize that one could optimize the interaction of compound **2**-like compounds with the subpocket and newly revealed active-site lid-open pocket. Potency gained by optimizing ligand binding in these hDAAO regions could conceivably reduce the need for extensive Arg^283^ association, and, thus, could facilitate the creation of novel inhibitors of hDAAO.

## Online data

Supplementary data
