# Supplementary material for: Novel human D-amino acid oxidase inhibitors stabilize an active-site lid-open conformation
Source: Biosci Rep. 2014 Aug 11;34(4):e00133. doi: 10.1042/BSR20140071 (PMC4127593; doi:10.1042/BSR20140071)
Supplement: Supplementary data [file bsr034e133add.pdf]

Biosci. Rep. (2014) / **34** / art:e00133 / doi 10.1042/BSR20140071

OPEN ACCESS

Original Paper

Bioscience Reports www.bioscirep.org

SUPPLEMENTARY DATA

Novel human D-amino acid oxidase inhibitors stabilize an active-site lid-open conformation

Ryan T. TERRY-LORENZO*[1], Lawrence E. CHUN†, Scott P. BROWN*, Michele L. R. HEFFERNAN*, Q. Kevin FANG*, Michael A. ORSINI*, Loredano POLLEGIONI‡§, Larry W. HARDY*, Kerry L. SPEAR* and Thomas H. LARGE*

*Discovery Research Department, Sunovion Pharmaceuticals, Marlborough, MA 01752, U.S.A.
†Emerald Bio, Bainbridge Island, WA 98110, U.S.A.
‡Dipartimento di Biotecnologie e Scienze della Vita, Universita degli Studi dell'Insubria, Via J. H. Dunant 3, 21100 Varese, Italy
§The Protein Factory, Politecnico di Milano, ICRM-CNR Milano and Universita degli Studi dell'Insubria, Via Mancinelli 7, 20131 Milano, Italy

EXPERIMENTAL

Designing the focused library

To prepare a library of compounds for screening we first filtered the then current (as of June, 2012) snapshot of the eMolecules database of commercially available compounds (http://www.emolecules.com; released March, 2012, and last accessed 22 January 2014). A set of molecular property filters (Table S1) were then applied to the eMolecules compounds (initially over 6 million compounds in total). This filtering reduced the total number of compounds to just over 320 000. This filtered set of eMolecules compounds was then used as input into three different computational methods: a 2D ligand-based method; a 3D ligand-based method; and a 3D structure-based method.

The ligand-based methods required a set of known active molecules. These were obtained directly from the protein–ligand structures listed in Table S2. This set of three compounds (the 'query' set) was used in all of the ligand-based searching on the filtered eMolecules' compounds (the 'database' set). The three query compounds shown in Table S2 were selected from the set of known DAAO actives by optimization of structural diversity (i.e. dissimilarity) using ECFP-6 (extended connectivity fingerprints of length 6) [1].

Before performing the 3D calculations it was necessary to generate 3D conformations for each molecule in both query and database sets. Omega (v2.4.6) [2] was used to generate plausible sets of conformations for each database molecule using the default settings for all command-line parameters, with the exception of the following: an energy window value of 50; a maximum number of conformers of 1000; and root-mean-square conformer-clustering threshold of 0.75 Å. For the query compounds, Omega was used with default settings for everything except the maximum number of conformers, which was set to 1.

To incorporate 3D structural information into our assessment of the database compounds, we used a docking and scoring approach. There were two primary reasons for introducing protein structure into the calculation: First, we were looking to bias our screening library for active-site similarity to obtain compounds more generally compatible with the full size and shape of the DAAO active site. Secondly, we sought to explore new regions of the binding site by requiring additional positional constraints on the docked poses during the docking procedure.

Table S1 Property cut-off values used to perform a first-pass triage of the full set of eMolecules compounds
All properties were calculated and filtered using Pipeline Pilot (v8.5.0.200) (Accelrys, http://www.accelrys.com; accessed 22 January 2014)

Property	Min	Max
Molecular mass	250	450
Hydrogen-bond donors	0	8
Hydrogen-bond acceptors	0	4
AlogP	− 0.5	4.5
Polar surface area	0	100
Rotatable bonds	0	8
Number of aromatic rings	0	4

In the following sections, we describe three different methods used to search the database compounds. In the final section, we describe the steps to combine the individual methods and produce the final prioritized list of candidate compounds for consideration in the screening library.

2D ligand-based calculations

To assess 2D ligand similarity against the query compounds, we used ECFP-6, which was calculated and processed within Pipeline Pilot (v8.5.0.200) with a bit length of 4096. The output from this was three separate rank-ordered lists of database compounds, one for each of the three query molecules. The compounds in each of these lists contain an ECFP-6 Tanimoto similarity value to each known active, the values of which ranged from 1.0 (identical) to 0.0 (orthogonal).

[1] To whom correspondence should be addressed (email Ryan.Terry-Lorenzo@sunovion.com).

© 2014 The Author(s) This is an Open Access article distributed under the terms of the Creative Commons Attribution Licence (CC-BY) (http://creativecommons.org/licenses/by/3.0/) which permits unrestricted use, distribution and reproduction in any medium, provided the original work is properly cited.

Table S2 Chemical structures for the three query compounds used in the ligand-based strategies, along with the accompanying truncated FAD fragment

Also listed in the table are the pdb sources of the 3D coordinates for each molecule.

Identifier	Structure	Source
Compound S-1	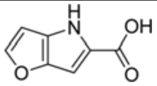	PDB: 3CUK
Compound S-2	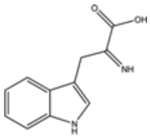	PDB: 1DDO
Compound S-3	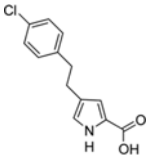	PDB: 3ZNO
FAD fragment	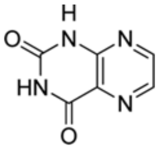	(3D coordinates taken from each of the above structures)

3D ligand-based calculations

The three queries shown in Table S2, along with their accompanying FAD fragments, were used to perform the 3D similarity comparisons. To compare 3D similarity we used the program ROCS (v3.1.2) [3]. The ROCS calculation returns shape Tanimoto and colour Tanimoto values for the single best conformer overlay of each database molecule onto each of the three query molecules. The full calculation also incorporates an accompanying FAD fragment that adds its shape and colour to each query molecule. The 3D coordinates for each FAD fragment were obtained from the cognate structure of each query molecule (specified in Table S2). From this step we generated a separate list of rank-ordered compounds for each query, which was sorted by the combined score of shape Tanimoto plus colour Tanimoto.

3D structure-based calculations

We used the protein coordinates from the three X-ray crystallographic structures (the cognate structures for each query) listed in Table S2. To prepare the protein structures for consistent handling in the scoring procedure, we added explicit hydrogen atoms to each complex using OpenEye Scientific Software's hydrogen-atom placement algorithm in OEChem (http://www.eyesopen.com/; last accessed 22 January 2014). All of the database compounds were then docked into each protein structure using FRED (v2.2.5) [4]. FRED broke down the process into three phases: (1) a grid was constructed that encompassed the region of the protein within which we wished to search; (2) the input conformations for each molecule were systematically translated and rotated on the protein active-site grid to produce an exhaustive set of putative binding poses, which were then triaged down to a manageable number by initial scoring; and (3), the top-scoring poses from (2) were passed into final score optimization to produce a single highest-scoring best pose for each compound.

To generate the grids in (1) we used the position of the crystal structure ligand to centre each grid 'box,' after which 4.0 Å was added to each side of the grid box. We added to each grid an additional pose filter that contained three constraints in the active site. The first two constraints placed two spheres with a radius 1.5 Å on FAD fragment atoms O2 and O4. The final constraint placed a 1.5 Å sphere on the atom labelled CZ in the protein active-site arginine residue (residue Arg^{283} in 2DU8). Any docked poses that could not place at least 1 atom of the correct functional group into each of these spheres was eliminated from consideration. Poses that survived these constraints were then scored in step (2) using CGO (Chemical Gaussian Overlay), which filtered out all but the top scoring 100 poses. The CGO score was chosen in order to provide a measure of similarity against the known ligands, which allowed for incorporation of additional bias towards experimental data. These poses were then optimized using ChemGauss3 [4] in step (3) of the docking. The above docking procedure produced three rank-ordered lists, one for each structure used.

Combining scores

To combine the information from all of the above methods we used a general framework based on Belief Theory [5] to provide a final net cumulative score. This requires all of the similarities from the ligand-based methods [6], as well as the docking scores from the structure-based method, to be converted into probabilities [7]. The individual probabilities, or 'beliefs,' from the each method were then combined in a formal way to create a total belief in the activity of each database molecule. We used equation (S1) to combine the probabilities for the ligand-based beliefs

© 2014 The Author(s) This is an Open Access article distributed under the terms of the Creative Commons Attribution Licence (CC-BY) (http://creativecommons.org/licenses/by/3.0/) which permits unrestricted use, distribution and reproduction in any medium, provided the original work is properly cited.

from ECFP-6 and ROCS, and the structure-based beliefs from FRED: (S1)

$$\text{Cumulative belief} = 1 - \prod_{i=1,j=1}^{N,M} (1 - P_{i,j}) \qquad \text{(S1)}$$

where N is the number of methods, M is the number of queries, and $P_{i,j}$ is the probability for the ith measure on the jth query. The cumulative beliefs produced by the conjunctive combination in equation (S1) were then used to produce a final ranked list of database compounds. The top several thousand compounds from this list were retained and passed for final evaluation by a project-team medicinal chemist, who then generated the final list of compounds to be screened.

Compound screening

The focused library of 1016 compounds was screened for hDAAO inhibition using the Amplex Red-based platform. Compounds were screened at 50 and 5 μM in parallel in the hDAAO inhibition and counter assays. Hits were considered compounds in which the% inhibition at 50 μM was >35 percentage points higher in the hDAAO inhibition versus the counter assay. These hits were further characterized by determining an hDAAO IC_{50}. To quantitatively determine assay performance, a Z' factor [8] was determined using standard protocols and equations. Briefly, hDAAO activity (based upon fluorescent product produced) was determined in 32 interleaved wells each of 3 conditions: DMSO-treated (Signal), 3.3 nM compound **1**-treated (Mid value), and 3.3 μM compound **1**-treated (background). The compound **1** concentration in the Mid value condition was chosen as an approximate IC_{50}, and the background concentration was a compound **1** concentration that produced 100 % hDAAO inhibition. This experiment was repeated using three plates each on three different days. The signal and background wells were used to determine a Z' Factor, a single metric that quantitatively defines the suitability of an assay for screening by accounting for assay signal, background and S.D. of both signal and background [8].

Synthesis of compound 3

The general synthetic scheme for compound **3** is described in Supplementary Figure S1. More details are below.

((2,4-dimethoxyphenyl)ethynyl)trimethylsilane

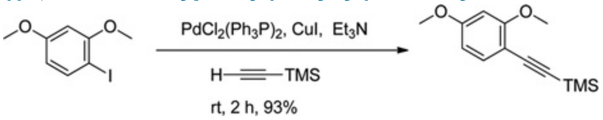

To a solution of 1-iodo-2, 4-dimethoxybenzene (1.32 g, 5.0 mmol), $PdCl_2(PPh_3)_2$ (0.07 g, 0.1 mmol) and CuI (0.02 g, 0.1 mmol) in TEA (triethanolamine) (20 ml) (stirring for 5 min beforehand), 6.0 mmol of trimethylsilyl acetylene (0.588 g, 6.0 mmol) in 5 ml of TEA was added dropwise over 10 min under Ar atmosphere. Then the mixture was stirred at room temperature for 1.5 h. The resulting solution was filtered, washed with brine and extracted with ethyl acetate (2 × 10 ml). The combined organics were dried ($MgSO_4$) and concentrated under vacuum to provide the crude product. Purification by flash chromatography to provide 1.1g (93 % yield) of ((2,4-dimethoxyphenyl)ethynyl)trimethylsilane as a brown solid. ^{1}H NMR (400 MHz, $CDCl_3$) δ 7.32 (d, J = 8.0 Hz, 1H), 6.36 (d, J = 8.0 Hz, 1H), 6.34 (s, 1H), 3.78 (s, 3H), 3.72 (s, 3H), 0.25 (s, 9H). MS (ESI) m/z 235 (M + H)$^+$.

1-Ethynyl-2,4-dimethoxybenzene

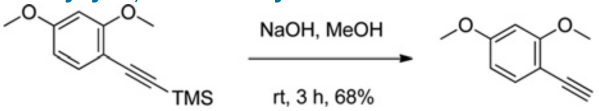

A solution of NaOH (1.60 g, 40.0 mmol) in 15 ml of water was added dropwise to [(2, 4-dimethoxyphenyl)ethynyl]trimethylsilane (2.34 g, 10 mmol) in 45 ml of 50 % of CH_3OH/diethyl ether. The reaction mixture was stirred for 3 h at 25 °C. CH_3OH was removed under vacuum. The reaction mixture was diluted with ether and neutralized with 2 M HCl. The ether layer was washed with water, dried (Na_2SO_4), filtered, and the solvent removed under vacuum. Purification by flash chromatography to provide 1.1 g (68 % yield) of 1-ethynyl-2,4-dimethoxybenzene as a clear oil. ^{1}H NMR (400 MHz, $CDCl_3$) δ 7.36 (d, J = 8.4 Hz, 1H), 6.42 (d, J = 8.4 Hz, 1H), 6.41 (s, 1H), 3.84 (s, 3H), 3.78 (s, 3H), 3.25 (s, 1H). MS (ESI) m/z 163 (M + H)$^+$.

3,4-Bis(benzyloxy)-6-chloropyridazine

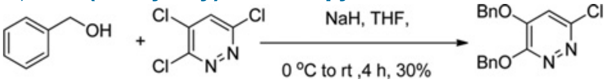

To a solution of sodium hydride (4.98 g, 124.4 mmol) in THF (200 ml) was added phenylmethanol (13.45 g, 124.4 mmol) at room temperature. The resulting mixture was stirred for 1 h and then cooled to 0 °C. 3,4,6-trichloropyridazine (11.41 g, 62.2 mmol) in THF (tetrahydrofolate) (50 ml) was then added dropwise. The reaction mixture was stirred at room temperature for 4 h. EtOAc (400 ml) was added to the reaction vessel and the resulting biphasic mixture was transferred to a separator funnel. The layers were separated and the organic phase was washed with saturated aqueous NaCl (1 × 75 ml). The combined organics were dried (Na_2SO_4), filtered and concentrated *in vacuo*. The resulting oil was purified by flash column chromatography with 20/1 PE/EA) to provide 6.2 g (30.5 % yield) of 3, 4bis(benzyloxy)-6-chloropyridazine as a white solid. MS (ESI) m/z 327.0 (M + H)$^+$.

3,4-Bis(benzyloxy)-6-((2,4-dimethoxyphenyl)ethynyl)pyridazine

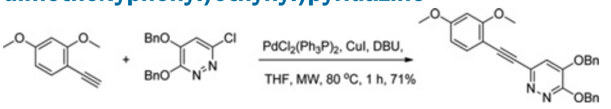

To a solution of 1-ethynyl-2,4-dimethoxybenzene (0.16 g, 1 mmol) in THF (5 ml) was added 3,4-bis(benzyloxy)-6-chloropyridazine (327 mg, 1 mmol) followed by copper iodide (0.02 g, 0.1 mmol), DBU (0.46 g, 3 mmol) and $PdCl_2(Ph_3P)_2$

© 2014 The Author(s) This is an Open Access article distributed under the terms of the Creative Commons Attribution Licence (CC-BY) (http://creativecommons.org/licenses/by/3.0/) which permits unrestricted use, distribution and reproduction in any medium, provided the original work is properly cited.

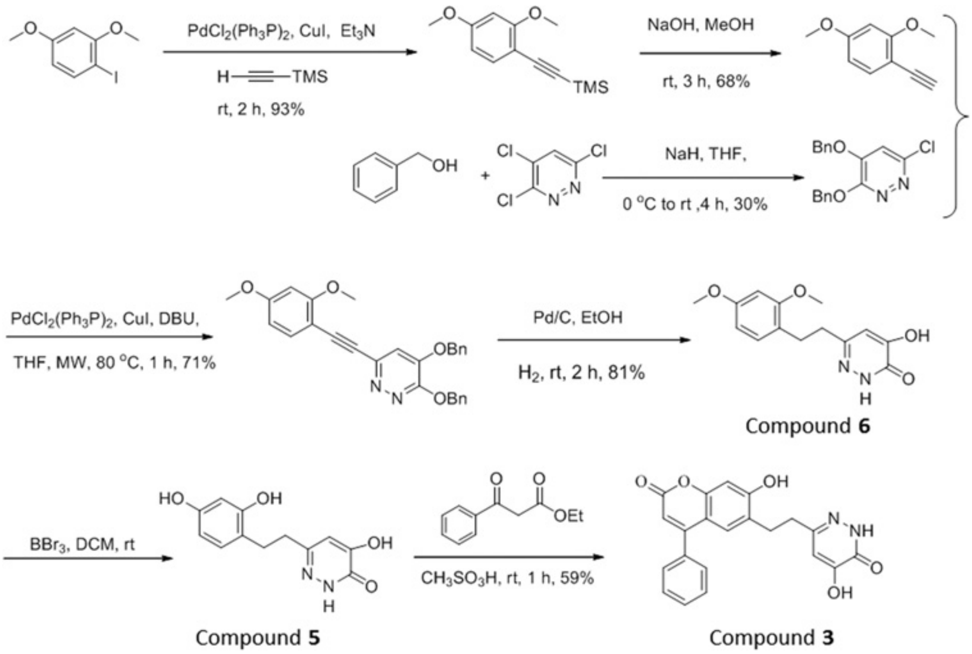

Figure S1 Chemical synthesis scheme for compound 3

(0.04 g, 0.05 mmol) in a 20 ml microwave vial. The mixture was purged with nitrogen before the whole mixture was subjected to microwave radiation for 1 h at 85 °C. The reaction mixture was cooled to room temperature and diluted with ethyl acetate and purified by silica gel chromatography to provide 320 mg (71 %. yield) of 3,4-bis(benzyloxy)-6-((2,4-dimethoxyphenyl)ethynyl)pyridazine as an orange oil. MS (ESI) *m*/*z* 453 (M + H) $^{+}$.

6-(2,4-Dimethoxyphenethyl)-4-hydroxypyridazin-3(2H)-one

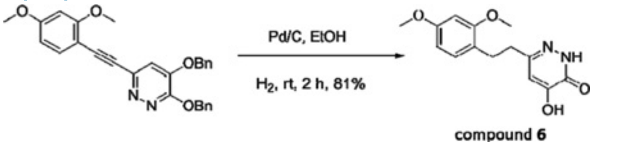

To a solution of 3, 4-bis(benzyloxy)-6-((2,4-dimethoxyphenyl) ethynyl)pyridazine **(1–7)** (230 mg, 0.05 mmol) in ethanol (10 ml) under nitrogen was added palladium (10 wt.% on activated carbon, 23 mg). The reaction mixture was deoxygenated under vacuum and then hydrogenated at room temperature for 2 h. After the reaction completed, the reaction mixture was filtered through a pad of celite and washed with ethnol (2 × 25 ml). The filtrate was concentrated to provide 114 mg (81 % yield) of 6-(2, 4-dimethoxyphenethyl)pyridazine-3,4-diol) as a brown solid. This crude product was used for the next step without further purification. ^{1}H NMR (400 MHz, DMSO) δ 12.64 (s, 1H), 10.69 (s, 1H), 6.99 (d, *J* = 8.3 Hz, 1H), 6.56–6.49 (m, 2H), 6.41 (dd, *J* = 8.3, 2.4 Hz, 1H), 3.75 (s, 3H), 3.72 (s, 3H), 2.77-2.71 (m, 2H), 2.68–2.61 (m, 2H). MS (ESI) *m*/*z* 277 (M + H) $^{+}$.

6-(2,4-Dihydroxyphenethyl)-4-hydroxypyridazin-3(2H)-one

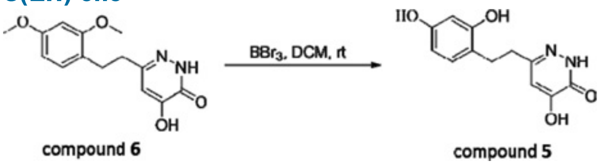

To a solution of a 6-(2, 4-dimethoxyphenethyl)-4-hydroxypyridazin-3(2H)-one (2 mmol) in dry CH_2Cl_2 (5 ml) was added dropwise BBr_3 (1.2 mmol, 0.3 ml) at -20 °C. Then the reaction mixture was warmed to room temperature and stirred at that temperature overnight. HCl in methanol (1 M, 2 ml) was added to quench the reaction at 0 °C. The mixture was concentrated under vacuum. The crude product was enough pure for the use of next step. ^{1}H NMR (400 MHz, DMSO) δ 12.64 (s, 1H), 10.67 (s, 1H), 8.85–9.25 (m, 2H), 6.77 (d, *J* = 8.2 Hz, 1H), 6.51 (s, 1H), 6.26 (d, *J* = 2.3 Hz, 1H), 6.10 (dd, *J* = 8.1, 2.4 Hz, 1H), 2.70–2.60 (m, 4H), 2.54 (s, 1H). MS (ESI) *m*/*z* 249 (M + H) $^{+}$.

4-Hydroxy-6-(2-(7-hydroxy-2-oxo-4-phenyl-2H-chromen-6-yl)ethyl)pyridazin-3(2H)-one

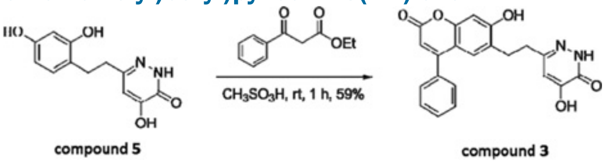

To a solution of 6-(2,4-dihydroxyphenethyl)-4-hydroxypyridazin-3(2H)-one (302 mg, 1.21 mmol) in methanesulfonic acid (5 ml) was added ethyl 3-oxo-3-phenylpropanoate (230 mg,

© 2014 The Author(s) This is an Open Access article distributed under the terms of the Creative Commons Attribution Licence (CC-BY) (http://creativecommons.org/licenses/by/3.0/) which permits unrestricted use, distribution and reproduction in any medium, provided the original work is properly cited.

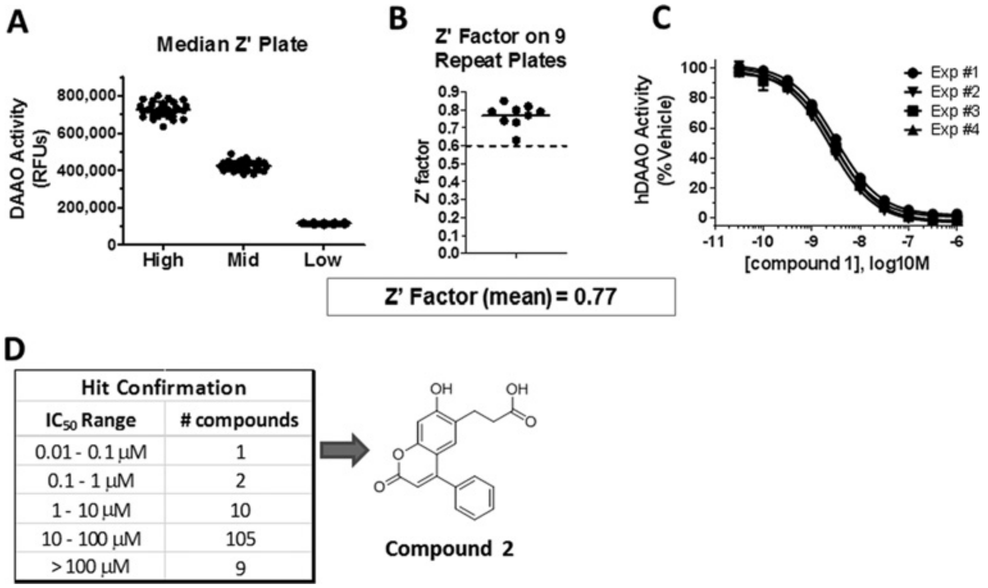

Figure S2 **Screening strategy identified compound 2**

(**A**) In the Amplex Red system in 96-well plate format, hDAAO activity, based upon RFUs (relative fluorescent units) of product produced, was determined in 32 interleaved wells each of three conditions: DMSO-treated ('High'), 3.3 nM compound 1-treated ('Mid'), and 3.3 μM compound 1-treated ('Low'). Shown in Figure 1(A) are results without normalization or background-subtraction from an average (median) plate. (**B**) On nine repeat plates (three plates each on three different days) of the experiment described in Figure 1A, the High and Low wells were used to determine a Z' Factor [8]. The Z' Factors on all nine plates were above 0.6, indicating that the assay was suitable for differentiating hDAAO inhibitors and non-inhibitors when screening compounds in single-dose format. (**C**) Inhibition curves with compound **1** in four independent experiments demonstrated assay reproducibility in reporting IC_{50} values. (**D**) 127 hits from the screen were tested for hDAAO inhibitory potency. Shown are the numbers of compounds in each histogram bin of inhibitory potency. Compound 2 was the most potent hit from the screen.

1.21 mmol).The reaction mixture was stirred at room temperature for 1 h. Water (50 ml) was added to the reaction vessel and the resulting solid was collected by filtration. The solid was purified by Pre-HPLC to provide 270 mg (59% yield) of 4-hydroxy-6-(2-(7-hydroxy-2-oxo-4-phenyl-2H-chromen-6-yl)ethyl)pyridazin-3(2H)-one (compound **3**) as a white solid. ^{1}H NMR (400 MHz, DMSO) δ 12.65 (s, 1H), 10.77 (s, 2H), 7.58-7.48 (m, 3H), 7.34 (dd, $J = 6.4$, 2.8 Hz, 2H), 6.94 (s, 1H), 6.84 (s, 1H), 6.52 (s, 1H), 6.10 (s, 1H), 2.79 (t, $J = 6.9$ Hz, 2H), 2.65 (t, $J = 7.0$ Hz, 2H). MS (ESI) m/z 377 (M + H)$^{+}$.

RESULTS

Identification of compound 2 as a novel inhibitor of hDAAO

To identify the novel inhibitors of hDAAO, we performed a series of modelling techniques to guide the purchase of commercially available compounds. These techniques ultimately produced a group of compounds modelled as bisubstrate analogues containing, in a single molecule, structural features of an active site hDAAO inhibitor adjacent to structural elements of the FAD cofactor. 1016 compounds were purchased and screened for hDAAO inhibition.

To screen these molecules for hDAAO inhibition, we used an Amplex Red-based fluorescence enzyme assay to measure the hydrogen peroxide produced during the hDAAO catalytic cycle of FAD reduction and re-oxidation [9]. The assay was robust with low S.D. and good signal-to-noise ratio, as evidenced by high Z' factor [8] values (Supplementary Figures S2A and S2B). hDAAO activity was inhibited potently and reproducibly by a known hDAAO inhibitor [10,11], compound **1** (Supplementary Figure S2C and Table 2). The screen of 1016 compounds was executed, using a counter assay to exclude compounds that interfered with the HRP/hydrogen peroxide detection system. For hit confirmation, 127 compounds that displayed >35% specific hDAAO inhibition at 50 μM were characterized in concentration–response experiments to generate IC_{50} values. The range of IC_{50} results are listed in Supplementary Figure S2(D). The most potent hit compound was compound 2.

REFERENCES

1 Rogers, D. and Hahn, M. (2010) Extended-connectivity fingerprints. J. Chem. Inf. Model. **50**, 742–754 CrossRef PubMed

© 2014 The Author(s) This is an Open Access article distributed under the terms of the Creative Commons Attribution Licence (CC-BY) (http://creativecommons.org/licenses/by/3.0/) which permits unrestricted use, distribution and reproduction in any medium, provided the original work is properly cited.

2 Bostrom, J., Greenwood, J. R. and Gottfries, J. (2003) Assessing the performance of OMEGA with respect to retrieving bioactive conformations. J. Mol. Graph Model. **21**, 449–462 CrossRef PubMed

3 Grant, J. A., Gallardo, M. A. and Pickup, B. T. (1996) A fast method of molecular shape comparison: a simple application of a Gaussian description of molecular shape. J. Comput. Chem. **17**, 1653–1666 CrossRef

4 McGann, M. (2011) FRED pose prediction and virtual screening accuracy. J Chem. Inf. Model. **51**, 578–596 CrossRef PubMed

5 Shafer, G. (1976) A Mathematical Theory of Evidence, Princeton University Press

6 Muchmore, S. W., Debe, D. A., Metz, J. T., Brown, S. P., Martin, Y. C. and Hajduk, P. J. (2008) Application of belief theory to similarity data fusion for use in analog searching and lead hopping. J. Chem. Inf. Model. **48**, 941–948 CrossRef PubMed

7 Swann, S. L., Brown, S. P., Muchmore, S. W., Patel, H., Merta, P., Locklear, J. and Hajduk, P. J. (2011) A unified, probabilistic framework for structure- and ligand-based virtual screening. J. Med. Chem. **54**, 1223–1232 CrossRef PubMed

8 Zhang, J. H., Chung, T. D. and Oldenburg, K. R. (1999) A simple statistical parameter for use in evaluation and validation of high throughput screening assays. J. Biomol. Screen. **4**, 67–73 CrossRef PubMed

9 Brandish, P. E., Chiu, C. S., Schneeweis, J., Brandon, N. J., Leech, C. L., Kornienko, O., Scolnick, E. M., Strulovici, B. and Zheng, W. (2006) A cell-based ultra-high-throughput screening assay for identifying inhibitors of D-amino acid oxidase. J. Biomol. Screen. **11**, 481–487 CrossRef PubMed

10 Sparey, T., Abeywickrema, P., Almond, S., Brandon, N., Byrne, N., Campbell, A., Hutson, P. H., Jacobson, M., Jones, B. and Munshi, S. (2008) The discovery of fused pyrrole carboxylic acids as novel, potent D-amino acid oxidase (DAO) inhibitors. Bioorg. Med. Chem. Lett. **18**, 3386–3391 CrossRef PubMed

11 Duplantier, A. J., Becker, S. L., Bohanon, M. J., Borzilleri, K. A., Chrunyk, B. A., Downs, J. T., Hu, L. Y., El-Kattan, A., James, L. C., Liu, S. et al. (2009) Discovery, SAR, and pharmacokinetics of a novel 3-hydroxyquinolin-2(1H)-one series of potent D-amino acid oxidase (DAAO) inhibitors. J. Med. Chem. **52**, 3576–3585 CrossRef PubMed

Received 30 April 2014/10 June 2014; accepted 1 July 2014

Published as Immediate Publication 8 July 2014, doi 10.1042/BSR20140071

© 2014 The Author(s) This is an Open Access article distributed under the terms of the Creative Commons Attribution Licence (CC-BY) (http://creativecommons.org/licenses/by/3.0/) which permits unrestricted use, distribution and reproduction in any medium, provided the original work is properly cited.
